# Grounding Language Processing: The Added Value of Specifying Linguistic/Compositional Representations and Processes

**DOI:** 10.5334/joc.155

**Published:** 2021-04-01

**Authors:** Pia Knoeferle

**Affiliations:** 1 Institut für deutsche Sprache und Linguistik, Unter den Linden 6, Humboldt-Universität zu Berlin, 10099 Berlin

**Keywords:** Embodied cognition, Sentence processing, Semantics, Eye movements

## Abstract

Abundant empirical evidence suggests that visual perception and motor responses are involved in language comprehension (‘grounding’). However, when modeling the grounding of sentence comprehension on a word-by-word basis, linguistic representations and cognitive processes are rarely made fully explicit. This article reviews representational formalisms and associated (computational) models with a view to accommodating incremental and compositional grounding effects. Are different representation formats equally suitable and what mechanisms and representations do models assume to accommodate grounding effects? I argue that we must minimally specify compositional semantic representations, a set of incremental processes/mechanisms, and an explicit link from the assumed processes to measured behavior. Different representational formats can be contrasted in psycholinguistic modeling by holding the set of processes/mechanisms constant; contrasting different processes/mechanisms is possible by holding representations constant. Such psycholinguistic modeling could be applied across a wide range of experimental investigations and complement computational modeling.

## 1 Introduction

Over the past three decades, many areas of cognitive science have taken up the idea that cognitive representations are ‘grounded’ or ‘embodied’ via perception and action. Among these are animal cognition (Gallese, Fadiga, Fogassi, & Rizzolatti, 1996), neuroscience (Chiel & Beer, 1997), artificial intelligence (e.g., Brooks & Stein, 1994; Steels & Brooks, 1995), philosophy (Clark, 1997), and language (Barsalou, 1999b, 1999a; Glenberg & Kaschak, 2002; Pulvermüller, Lutzenberger, & Preissl, 1999; Rizzolatti & Arbib, 1998). I will use ‘grounded’ and ‘embodied’ interchangeably to indicate the involvement of systems used for perception and action in the interpretation of language (for examples see below). For language, a central question has been to what extent lexical-semantic meaning overlaps with representations from visual perception and action and to what extent visual perceptual and action representations are essential for understanding the meaning of words (see Meteyard, Cuadrado, Bahrami, & Vigliocco, 2012, for an overview of positions from strong, weak, secondary, to non-embodied representations). Evidence for embodied language processing comes from a range of behavioral and neuroscientific measures (see, e.g., Buccino et al., 2005; Pulvermüller, Härle, & Hummel, 2001; Tettamanti et al., 2005). As one example, Pulvermüller et al. (2001) recorded high-resolution electrical brain activity as participants rapidly decided whether a written stimulus was (vs. wasn’t) a word. Participants’ response latencies in that task were shorter for action words involving the face than the lower body. Moreover, electrical brain activity reflected the activation of the brain areas controlling leg movements for verbs semantically related to leg actions (e.g., walking) from 250 ms after written word onset (p. 158). These and other results were interpreted as supporting an embodied view of lexical representations in which “words are cortically represented by cell assemblies whose topographies reflect the words’ lexical meaning” (p. 163).

However, “a close look at the literature reveals that the debate about the nature of the processes involved in language comprehension is far from settled, and key questions remain unanswered” (Ostarek & Huettig, 2019, p. 593). It has been argued that the field should transition from asking whether language is grounded to asking “when and how sensori-motor cortices play a role in understanding.” (Willems & Francken, 2012, Article 582, p. 2). Related arguments have highlighted that it is important to examine when and how “compositional aspects of language processing, such as tense and temporal cues” (Knoeferle, Crocker, & Pulvermüller, 2010, p. 140) modulate grounding (see also Knoeferle & Crocker, 2007, p. 542). Kaschak and Glenberg (2000) pointed out the need for compositionality even earlier: “Thus the evidence supports a specific type of interaction between syntax and semantics that leads to understanding: The syntax specifies a general scene, and the affordances of objects are used to specify the scene in detail sufficient to take action.” (p. 508).

Regarding compositionality, effects of grounding are at least partially sensitive to constituent order and its semantic interpretation. This sensitivity suggests that in accommodating effects of grounding, we must pay attention to representations beyond the lexical level and their incremental interaction. In the present article I focus on using symbolic representational formalisms (see Harnad, 1990 for a review) to accommodate incremental and compositional grounding effects. I review representative frameworks and models of grounded and situated language (processing), and assess their potential for modeling (a subset of) incremental and compositional effects of grounding. On the basis of the review, I argue that it would be advantageous to better specify linguistic representations, their incremental construction, and their link to dependent measures prior to data collection.

### 1.1 Evidence for grounding in comprehension: Incremental and compositional

Extant approaches do not yet offer a detailed description of the incremental grounding processes and compositional representations implicated in recovering the interpretation of a sentence (see Appendix A1 for details). But such a description is warranted: Psycholinguistic evidence suggests that grounding effects implicate compositional representations, and that action-based representations interact fairly incrementally and compositionally with visual attention and comprehension. Below, I review two studies in support of this view.

For a spoken sentence such as *The student will stagger along the trail to the picnic basket*, staggering implies slow motion. By contrast, the same sentence with a different verb like *run* implies fast motion. To the extent that verb-implied motion affects visual attention and comprehension, participants listening to these utterances should exhibit distinct gaze pattern on a related image. An image showed, for instance, a man, a trail, and a picknick basket. These objects functioned as parts of an event (an agent, a path and a goal) with the expectation being that verb speed, if re-enacted via eye movements, might influence how comprehenders attend to the goal and the path. Participants in the experiment by Lindsay, Scheepers, and Kamide (2013) on average looked more often and longer at the trail during *along the trail* following *The student will stagger* than *The student will run*. By contrast, for sentences containing *run*-verbs (compared with slow-motion *stagger*-verbs), participants looked earlier to the goal as they listened to the verb (see also Spivey & Geng, 2001; Kamide, Lindsay, Scheepers, & Kukona, 2016; Speed & Vigliocco, 2014, for related evidence). Time curve graphs and analyses of looks showed that these effects emerged during and just after the verb. Knowledge of motion speed made available by the verb in sentence context thus incrementally modulated a motoric, eye-movement response. That response implicated a (compositional) link between representations of verb speed and the path and goal representations of an event in spoken language processing.

Incremental and compositional grounding effects were also reported for reading, in relation to manual responses (Zwaan & Taylor, 2006). In a self-paced reading task, participants rotated a knob five degrees to advance from one sentence segment to the next. The sentences they read implied a clockwise (e.g., closing a bottle) or counter-clockwise (e.g., opening a bottle) direction of action by a mentioned protagonist (e.g., a runner). Reading times at the verb were reduced when the verb-implied rotation direction matched (vs. mismatched) the knob-rotation.

Taylor and Zwaan (2008) replicated this finding and showed that within a sentence (e.g., *The runner/was very/thirsty./A fan/handed him/a bottle/of cold/water/which he/opened/quickly*), the rotation mismatch effect was localized on *opened* and the ensuing adverb. Moreover, the meaning of the adverb mattered. When a postverbal adverb kept the focus on the action (e.g., *slowly* or *quickly*), congruence effects in reading times lasted into the adverb. But if the adverb shifted focus to the agent (e.g., *obediently* or *eagerly*), the effect emerged only at the verb, not the ensuing adverb. In addition to showcasing the incrementality of language grounding, these results also emphasize its compositional nature. Taylor and Zwaan (2008) suggested that language grounding during processing persists as long as the action is within linguistic focus. Once that focus shifts (e.g., from the action to an agent), grounding effects are predicted to come to an end.

In brief, evidence for incremental effects of non-linguistic information (knowledge of motion speed, the direction of manual rotation) emerged during spoken comprehension and during reading. The locus of the effects was temporally coordinated with – and dependent upon – sentence interpretation beyond lexical-referential processes (see also Bergen & Wheeler, 2010; Crocker, Knoeferle, & Mayberry, 2010; Guerra & Knoeferle, 2014; Huette, 2016; Kaup, Lüdtke, & Maienborn, 2010; Zwaan, Taylor, & Boer, 2010). Accommodating the compositionality and incrementality of such grounding in sentence comprehension requires a relatively detailed representational and procedural model.

## 2 Assessing models of language grounding

Given the evidence in favor of incremental and compositional grounding, implicating the conceptual level and aspects of meaning that reach into motoric representations/processes, we can assess models of language (processing) against the following benchmarks:

1. **Compositionality:** How/to what extent are language representations related to visual perception or action beyond the lexical level?
  - What level(s) of linguistic representation are grounded?
  - Are representations implicated in attention and manual responses (and quantifiable linking hypotheses) included?
2. **Incremental processes:** How/to what extent are compositional grounding effects modeled incrementally?
  - Is the timing of grounding effects made explicit?
  - What mechanisms are assumed?

Together these benchmarks help assess to what extent and how the available models ground language processing. Could they, in principle, accommodate the target findings by Lindsay et al. (2013), and Taylor and Zwaan (2008) among others? I have, for the most part, limited the review below to approaches that explicitly connect language to other cognitive subsystems and have selected representative accounts instead of providing an exhaustive discussion of all extant models. The selection of to-be-discussed approaches was motivated by the desire to consider both grounding of conceptual and of syntactic representation levels; by the desire to consider more than just one kind of grammar-based approach; and by the attempt to also assess models that can offer a linking hypothesis from conceptual representations to human behavior. The benchmarking regarding compositionality (section 2.1) and incrementality of grounding (section 2.2) will be followed by a synthesis and recommendations for how progress can be made (section 3).

### 2.1 Compositional grounding of representations

Both linguistically-motivated symbolic and non-linguistic representations have been grounded in relation to visual perception and action and offer representational compositionality.^1^ I assess these models against the compositionality-of-grounding benchmark (1) with a focus on points (a) and (b). Among the linguistic approaches, both Jackendoff and Construction Grammars (CG) connect linguistic representations via semantic/conceptual structure to representations derived from the non-linguistic context and these representations go beyond the lexical level (e.g., linking sentences to events). For instance, symbols like [MOON] stand for nouns like *moon*; features like [+round] can help enrich the noun’s meaning, and propositions capture compositional relations like *The rocket races to the moon* ([*_Event_ RACES* ([*_Thing_ ROCKET*], *_Path_ TO* ([*_Thing_ MOON*])], Jackendoff (2002)). In Jackendoff, syntactic representations can be paired with conceptual representations and these can interact with spatial/motor structures. That pairing of structures can be viewed as related to cognitive linguistics (see Goldberg, 1996). And indeed, other cognitive linguistic accounts feature compositional pairing of form and meaning with representations of the non-linguistic environment: Embodied Construction Grammar (ECG, Bergen & Chang, 2005) assumes cognitive schemas^2^ derived from perceptual and motor experience. For instance, one construction could link the phonological form of a cat to its schema and instances of cats (see Figure 10.1 in Bergen & Chang, 2013). The embodied (and other) construction grammar formalisms also ground compositionality (verb forms, for instance, can unlock schemas specifying grammatical function and associated event roles, as well as Execute (X)-schemas relating language to the world, e.g., Figure 16, the toss schema, and the X-schema for tossing in Figure 20 in Bergen and Chang (2005)).

One distinction in grounding between these cognitive linguistic approaches and other, minimalist grammar accounts is the level of linguistic representations that is linked to representations of visual perception and action (conceptual/semantic structure in cognitive linguistics versus syntactic representations in the minimalist grammar account by Knott (2014)). Knott (2014) draws on work linking cognitive processes and action planning to eye movements (Ballard, Hayhoe, Pook, & Rao, 1997). Just as Minimalism assumes building blocks for syntactic structure, Ballard et al. assume building blocks for sensorimotor processes. Knott exploits this analogy and grounds the recursive linguistic structure in Chomskyan grammar via acted-out eye movements.^3^

Representations of an immediate visually-perceived environment are thus explicit in the cognitive and minimalist grammar accounts; an explicit model of attention is central in Knott’s minimalist account, and links to visual attention are assumed in cognitive linguistics.^4^ But grounding of knowledge seems more easily accommodated when it occurs via conceptual structure than logical form since the latter does not encode the directionality of a bottle-opening action, and the speed and manner of motion implied by *run* vs. *stagger* in relation to further event representations.

Both representations of the non-linguistic environment and compositionality have also been included in models relying on distributed representations: Zeros and ones are used in connectionist networks to code features such as whether a representation stands for a noun (vs. verb), two different word orders, or case marking. This coding permits a computational network to process input representations via some function, and to generate output activation values that can be mapped into symbolic linguistic representations. In the Coordinated Interplay Account Network (CIANet), Mayberry et al. (2009) created random binary vectors to represent nouns and verbs (linguistic input) and the same vector representations also represented actions and actors (scene input, p. 461). Compositional grounding in the network came about through learned associations between vector representations of sentence structure and verbs with representations of scene events; these associations were mediated via an attentional mechanism (another vector). It is difficult to compare a connectionist model with linguistic symbolic approaches: But considering that the vectors are assumed to represent conceptual-level (event) representations, grounding in this connectionist model is arguably more akin to the grounding in the reviewed cognitive linguistic than minimalist approaches. The inclusion of an explicit attention mechanism is shared with the minimalist approach, but CIANet does not assume direct grounding of recursive syntactic representations in eye movements.^5^

Representations of attention, and of an immediate visually-perceived environment are, by contrast, not explicit in distributed situation space (DSS, Frank et al., 2003). But in DSS, representations of story content can be linked with representations of experience in a micro-world, including compositional relations. For instance, for *Jilly is outside*, a “1” is assigned if she is outside, while “0” marks not outside. Probabilities resulting from such individual instances can be rendered in a vector and capture the reader’s belief of how likely it is for Jilly (or any other object) to be outside (Frank et al., 2003 p. 881). Venhuizen, Crocker, and Brouwer (2018) exploit propositional logic more fully to represent events (*enter(beth, restaurant)*, p. 9) in a micro world and a mini grammar, representing both as vectors. Compositionality in language and world knowledge is captured via compositionality of propositions. Much of the appeal of DSS lies in its quantifiable link between vector representations, propositional representations reflecting states and events in a micro world, and human attention.

In brief, accounts that achieve linking via a compositional representation at the conceptual/semantic level, including event representations appear to more easily capture the representational grounding required to accommodate the target findings. Not all of the approaches feature an explicit model of attention, and they differ in how directly they map visual attention to syntactic and semantic interpretation. Of the approaches, most seem to model action execution in a third-person view (a man grabbing a cup, a rocket going to the moon, Beth entering a restaurant), as well as (visual) attention of the language user, but not his/her manual responses (the dependent measure in Taylor and Zwaan (2008); but see Knoeferle, Urbach, and Kutas (2014) on including verification response times in a processing account of situated language).

### 2.2 Incremental processes

To model the time-course of the target findings (e.g., Lindsay et al., 2013; Taylor & Zwaan, 2008), we must accommodate incrementality at approximately the word-level. Many of the reviewed accounts achieve this; however, not all models specify the time course of grounding effects in a principled manner, and they differ in the implicated mechanisms.

**Incrementality** is absent in the Jackendoffian account of language; but the timing of grounding effects is made explicit in the Coordinated Interplay Account (Knoeferle & Crocker, 2006, 2007), for which the assumption is that grounding effects emerge closely temporally coordinated with words or phrases that elicit them. Computational implementations of Construction Grammar can also capture incrementality. Embodied Construction Grammar, for instance, envisages incrementality and sense disambiguation (Section 3 in Bergen & Chang, 2005) (see also Bryant, 2008, on incremental reading time data). However, at what point during comprehension an Execute(X)-schema is activated would need to be determined in a principled manner. Its activation could be mediated by the verb *run*, or by the agent *the student* (if s*he were perceived as running), or later, if this process is assumed to take some time. For accommodating reading-time data, Bryant (2008) assumes that constructions are context-independent (Bryant, 2008, p. 56, equation 4.3). The model contains only a limited representation of scene context with a view to improving reference resolution (Bryant, 2008, p. 209 ff.). For grounding effects that implicate language-based and world knowledge this poses no problem and reference resolution appears also covered. To accommodate incremental grounding beyond reference resolution, the context representation would likely need to be extended and the timing specified (p. 187f.). What Bryant’s model includes, however, is a linking hypothesis between model probabilities and reading times, via surprisal, meaning that the timing of grounding effects can be specified (see Hale (2003); Bryant, 2008, p. 174ff.). Incrementality is absent in minimalist grammar (Chomsky, 1995) but present in Knott’s linking of logical form to sensorimotor processes. That link permits accommodating the incremental grounding of sentences like *The man grabs the cup*. Knott assumes real-time interaction of grammatical representations with deictic representations (acquired via eye movements). Incrementality of grounding language comprehension in relation to attention in a scene is captured by connectionist models (e.g., Mayberry et al. (2009); Kukona and Tabor (2011); for modulation of speech recognition by visual context see Roy & Mukherjee, 2005). Regarding incremental grounding, Kukona and Tabor (2011)’s approach can, for instance, capture looks to referents and to semantically related objects (for empirical evidence see Huettig & Altmann, 2005, Kukona et al. (2011) p. 24 for discussion of modeling work). Incrementality is also present in a sentence processing model that employs Distributed Situation-Space representations Venhuizen et al., 2018). It models the integration of world knowledge with language and can make quantitative predictions regarding attention and processing difficulty.

**Mechanisms** with the functionality of reconciling scene and language (i), and of attentional guidance (ii) are included in most of the models: In the implemented FUSE model, Roy and Mukharjee (2005) accommodate how speech recognition and scene contents are “fused” (p. 227): Objects in a scene receive attention via a dynamic attention mechanism if their mention is likely given the speech input (but see p.1041f. in Kukona & Tabor, 2011). Timing is thus made explicit. The same holds for Kukona and Tabor (2011) who rely on an increase in activation (‘pulse’) of nodes (standing for objects) in their network and liken the latter to attention (p. 1018; the authors also argue the model implicates learned associations between gaze behavior, linguistic, and visual context, p. 1040). An incremental attention mechanism (implemented via a gating vector) is featured in the Coordinated Interplay Account Network, too (‘CIANet’) (Mayberry et al., 2009). The attention vector acts as a gate and boosts language-matching (vs. mismatching) event representations; the grounding of the sentence interpretation against the event representations occurs incrementally, word-by-word, and anticipatorily (e.g., event depictions can elicit expectations). In corresponding psycholinguistic models, a co-indexing mechanism (linking structures in Jackendoff’s framework) has been used to support scene-sentence mapping in real-time language processing (see the Indexical Hypothesis by Glenberg and Robertson (1999), Knoeferle & Crocker, 2006, 2007)^6^. As a central mechanism, the Coordinated Interplay Account (CIA, Knoeferle & Crocker, 2006, 2007; Knoeferle et al., 2014) also assumes that attention is guided by an incrementally unfolding interpretation and associated expectations over a (representation of a) scene. Once attentional grounding has taken place, co-indexing and reconciliation of individual instances of language- and scene-derived representations takes place.

An attention mechanism plays a role in Knott’s account and in CIANet (gating vector), as well as in the CIA, together with co-indexing and reconciliation. Knott (2014) in addition assumes a simulation mechanism as instantiated via the linking of recursive syntactic with sensorimotor routines (p. 11, Proposal 1). This linking could be viewed as a reconciliation.^7^ Distributed Situation Space (DSS) and Embodied Construction Grammar both also feature reconciliation mechanisms, and ECG – much like Knott’s model, albeit differently thought-out, in addition features a simulation mechanism. Bergen and Chang (2005) rely on a unification mechanism for combining constructions and specifying an interpretation in context; they further assume that grounding of language occurs via a simulation mechanism, that can produce inferences (see section 3.2 in their chapter). Distributed situation-space (Venhuizen et al., 2018) has been implemented in a network that maps a localist word representation into a situation vector in DSS. The approach does not contain an explicit visual attention model that is guided by language interpretation like some of the other approaches. But a link to attention is present. From situation model probabilities, surprisal values are computed incrementally (after processing a given word, and in the context of the previous linguistic and micro-world context). The surprisal values are inversely proportional to word expectancy in context, as reflected in reading times, for instance (thus implicitly modeling attention and also explicitly specifying the timing of grounding effects). Surprisal has been linked to the incremental effects of discourse-level event representations (Metusalem et al., 2012), and event knowledge effects on verb complement expectations (Bicknell, Elman, Hare, McRae, & Kutas, 2010) among others but grounding effects like the target findings have not been explicitly modeled.

In summary, attention as a mechanism seems key in most accounts and helps in grounding language guided by the unfolding speech and semantic interpretation (FUSE, impulse processing, CIANet, CIA); attention is even tightly linked to recursive syntactic structuring (Knott, 2014); or it is predicted by the experience of language in situations (DSS). Only ECG and Knott assume a simulation mechanism. But similarities in mechanisms emerge functionally, for reconciling language with representations of a situation: In some models this is achieved via co-indexing or unification (e.g., CIA, ECG); in others via direct linking of syntactic and eye-movement recursion (Knott); in others by mapping localist word to distributed situation representations (DSS). As a linking hypothesis from comprehension to behavior, surprisal emerged as an interesting option.

## 3 Synthesis and suggestions for progress

This article assessed selected representational formalisms and (computational) models via two benchmarks: grounding compositional language representations (1) and the incrementality of grounding effects, as well as implicated mechanisms (2).

### 3.1 Compositional and incremental grounding of comprehension

Did some representational formalisms or models fare better than others when assessed against the benchmarks in section 2? All captured compositionality of representations in language. But none of the discussed approaches seemed to feature all of the representations required to accommodate the compositional semantic grounding suggested by the target findings. Most approaches lacked representations implicated in a comprehender’s manual response (and merely assumed links to manual action), and some did not feature a representation of the comprehender’s explicit (object-directed) attention (e.g., DSS). All except one (Knott’s minimalist) model seem to assume that grounding occurs via compositional representations at the conceptual, interpreted level of language. At that level, meaning distinctions of action speed and directionality of movement can be captured.

These differences in grounding notwithstanding, interesting convergences emerged for linking hypotheses (Construction Grammar and formal semantic propositions in distributed situation state space). Bryant (2008) and Venhuizen et al. (2018) combined embodied construction grammar and propositional semantic representations respectively with a probabilistic approach. Surprisal values computed from probabilities were linked to the incrementally-built Construction Grammar/propositional representations, and to human behavior. This added link translates semantic and situation representations into measurable human performance, something that could be added to other grammar formalisms that attempt to ground language in visual and motor representations.

Incremental grounding of language was modeled by many accounts and functionally most of these included mechanisms of attention and of reconciling language-based representations with representations of situation experience or of an immediate scene. Differences emerged at the level of realization of the mechanisms: attention-mediated co-indexing and reconciliation/verification (Knoeferle & Crocker, 2006, 2007; Knoeferle et al., 2014) versus simulation mechanisms (Bergen & Chang, 2005, 2013; Knott, 2014); direct analogy of recursive structure in language and eye-gaze (Knott, 2014), and direct relations of vectors derived from language and situations (Venhuizen et al., 2018). Some models were limited to spoken language and inspection of objects in context (Knott 2014)); others to story reading without visual attention to objects, (Venhuizen et al., 2018). Others capture both incremental grounding in reading and spoken comprehension (Knoeferle et al., 2014) but omit any simulation mechanism.

### 3.2 Suggestions for progress

Below I motivate and give an example of a first step towards progress: by making explicit how incrementally grounded compositional linguistic representations are constructed. One might argue that the added specification of linguistic representations cannot contribute in a meaningful way to the (computational) modeling of grounding language. After all, computational models make assumptions about grounding explicit; specifying linguistic representations would on this view have little added benefit. One might also be concerned that adding linguistic representations as another layer would not be fruitful since comparing connectionist with symbolic/linguistic representations is not straightforward (e.g., Smolensky, 2001; Steedman, 1999).

**Why specify grounded linguistic representations incrementally?** The present paper takes the stance that the combined specification of different models (linguistic representations and computational implementation) offers added value over specifying only one of these. Perhaps linguistic and non-linguistic representational formats are best viewed as complementary levels in linking cognition to behavior (see Smolensky, 2001, p. 324), even if the link is not straightforward. Connectionist models receive vector representations consisting of zeros and ones, associate them with other vector representations, and following such associative learning, output vector activation values. The representations implicated during processing are not stipulated but emerge (at hidden layers and via connection weights).

Granted, the output vectors can be associated with a symbolic compositional interpretation. But associating output vectors with a limited set of linguistic representations means that we see the output of grounded comprehension in linguistic notation but not how representations that we can reason about are incrementally grounded. With symbolic linguistic representations, we can reason about how a sentence interpretation is derived, what meaning is decoded, and how it is linked to representations of visual perception and action. Tabor (2009) argued that symbolic and connectionist approaches are incompatible (in line with Fodor and Pylyshyn (1988) but that this conflict can be resolved by appealing to non-linear dynamic approaches. In that spirit, Venhuizen et al. (2018) directly map propositional representations into situation vectors, and effectively bridge compositional semantic representations into vector space. Such formal direct mapping is one way to go; another option is to relate steps in a symbolic model with layers in a connectionist model and develop both in parallel but separately. This parallel comparison makes it easier for a wide range of scientists to contribute towards psycholinguistic modelling (effectively it sidesteps the bottleneck of limited computational training).

**Figure 1:**
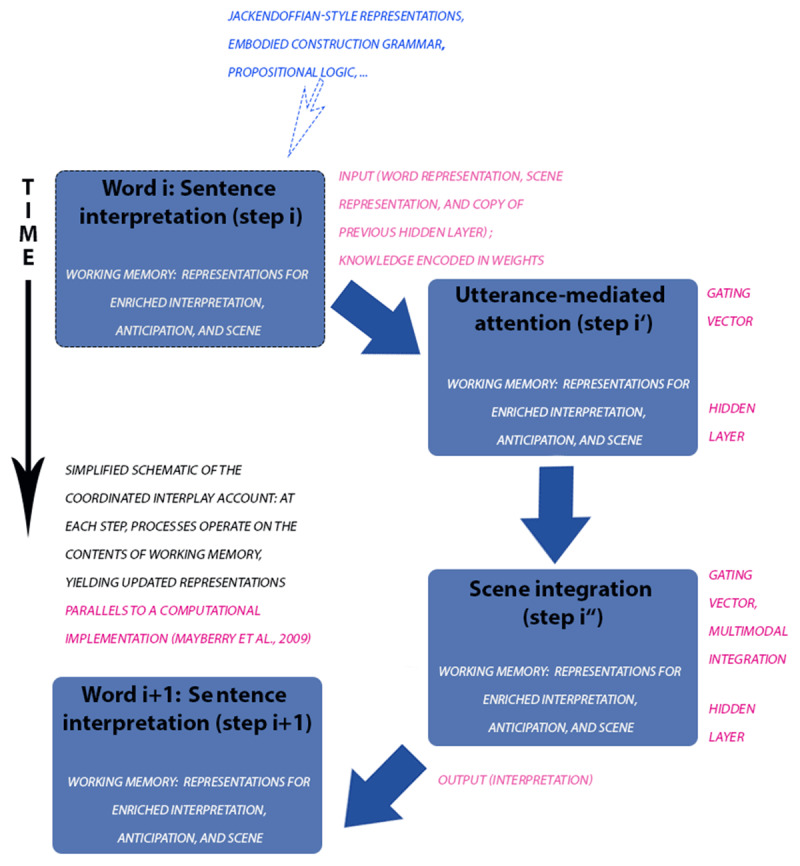
Simplified overview of the Coordinated Interplay Account (CIA) and its relation to a connectionist model (CIANet), Mayberry et al. (2009). Bright blue font: the CIA could be combined with different kinds of symbolic representations (these would be plugged in at step i as linguistic and world knowledge). Pink font: The account can be related to – and complemented by – predictions resulting from computational models. In addition to contrasting representations, one could keep these constant, and instead compare distinct mechanisms like simulation and verification (not depicted).

**How can we concretely make progress?** Only a small set of empirical findings on language grounding has been modeled computationally and scaling models can be a challenge. One possibility is to complement computational implementations by relating the process of constructing a grounded compositional interpretation (made explicit in linguistic representations) to steps in a computational model. ***Figure 1*** illustrates how the Coordinated Interplay account can be related to – and complemented by – computational modeling (pink font). Specifying linguistic representations and processes could lay the foundation for predicting grounding effects broad coverage (e.g., for a wide range of world-language relations), something that would constitute a substantial psycholinguistic contribution to the computational modeling of language grounding. Any skilled (psycho)linguist familiar with a relevant formalism could engage in this process, and if pursued this would lead to broad coverage hypotheses (and more formal linguistic interpretation) of grounding effects in language processing (across different sentence structures, their contexts, and languages, in younger, and older, in mono- and bi-lingual language users, among others).

If we adopt a processing framework (one example is given in ***Figure 1*** but others could be adopted instead), we could enrich it with linguistic representations from different grammar formalisms (or simplified linguistic representations derived from these), and compare how substituting one representation format with the other affects model predictions. ***Figure 1*** illustrates that the Coordinated Interplay account could be combined with different kinds of symbolic representations (these would be plugged in at step i as linguistic and world knowledge, indicated in blue font). To the extent that replacing one representation format with another does not change the predictions, one could use them in free variation. By contrast, better fit of predicted to observed response for one (vs. another) representational format across a substantial range of studies would lead to adopting that format (or aspects of it). Another possibility for making progress would be to keep the linguistic representations fixed but to contrast different mechanisms within a processing framework (e.g., a simulation versus verification mechanism).

Looking towards the future, further progress could be made by explicitly including speaker and listener characteristics into a processing account of language grounding. A recent extension of the CIA has done just that (Münster & Knoeferle, 2018). Thus extended, the CIA accommodated effects of speaker and listener characteristics. For instance, a speaker’s voice can rapidly interact with a listener’s world knowledge, affecting her comprehension (e.g., *I will drink some wine* spoken in a child’s voice does not fit with knowledge that children typically do not drink wine, and modulated event-related brain potentials within a few hundred miliseconds, Van Berkum, Brink, Tesink, Kos, & Hagoort, 2008). This rapid interaction, among others, can be captured by the extended CIA.

In summary, to make progress we want to (a) specify the grounding of language incrementally via linguistic representations (including of the language user) as we formulate hypotheses and interpret experimental results, complementing computational modeling; (b) contrast the assumed mechanisms and representation formats explicitly (hold one of these constant, vary the other).

## Footnotes

Among the approaches that employ linguistically-motivated symbolic representations, we can count Jackendoff’s theoretical framework of the language faculty (Jackendoff, 2002), the minimalist syntactic representations in Knott (2014), and construction grammar formalisms like Fluid Construction Grammar (Steels, 2017), Embodied Construction Grammar (Bergen & Chang, 2005), and Template Construction Grammar (Arbib & Lee, 2008). Among the approaches using vector representations to ground language comprehension, we can count connectionist modeling (e.g., Mayberry, Crocker, & Knoeferle, 2009) and distributed situation space (Frank, Koppen, Noordman, & Vonk, 2003) though the latter uses, in addition, propositional representations.

A ‘schema’ is defined as a representation that generalizes over individual instances of a thing. The non-linguistic schemas in Embodied CG are image schemas of Lakoff and Johnson (1980) and a symbolic variant of motor schemas, X-schemas (relating language to the world).

Linguistic details: For instance, linguistic representations of *The man grabs the cup* assume structure for *the man*, and nested below that for *the cup* and the verb *grabs*. Paralleling that, on the sensorimotor side, Knott’s account postulates that attention first goes to the man as the agent, with sensory feedback in the form of a representation of the man; attention next goes to the target, the cup, and in a third cycle, to the action of grabbing (Knott, 2014, p.11, p. 13ff.). Knott (2014) assumes that eye movements can reflect the recursive nesting of logical form within the preceding context and that each constituent in an X-bar schema has a sensorimotor interpretation (p. 11). For instance, in logical form an XP can be decomposed into Spec and to its right an X’. That X’ can in turn be decomposed into X and Comp. Linking to sensorimotor processes, the XP in logical form can be linked to an initial context resulting from a deictic operation; the X to a deictic operation, and the specifier to a re-afferent signal of that operation. The complement then relates to a new context created via the operation (Knott, 2014, p.11).

Knott (2014) assumes that motor actions like touching an object engage the agent’s tactile sensors which can result in adding deictic representations (p. 9). Bergen and Chang (2005) illustrate a schema for a jumping action (Figure 2 in their chapter), and analogously, modeling hand motion direction would appear possible.

A gating vector can boost attention to the verb-associated event out of two scene events, with both event and case-marking representations conveying the thematic roles of the event participants. Via these mechanisms (learning associations and gating attention), the network was able to predict an upcoming thematic agent in the output.

See Altmann and Mirković (2009) for a grounding mechanism that assumes overlap of representations.

For instance, when producing a sentence about a man grabbing an object, Knott (2014) assumes that an object is first allocated attention, and then hand actions ensue, creating a cross-modal representation; he also assumes that logical form structure is aligned in a cyclic fashion with deictic, eye-gaze routines, and can be replayed, suggesting incrementality (p. 17 and section 8.1). This linking has been implemented in a neural network model (Takac, Benuskova, & Knott, 2012) in which an interface network takes input from the current sentence context and from planned deictic routines (creating object representations), and outputs to a model performing a next-word production task (see Figure 9 in Knott, 2014).

## Appendix

### A1 Contribution of extant accounts

Many reviews have focused on predicting the extent of grounding effects rather than their timing and the construction of sentence-level linguistic representations. Barsalou (1999b) presents a proof of concept that perceptual symbol systems can accommodate key language characteristics much like amodal systems (e.g, propositions, productivity, and abstract concepts, p. 577, 581, 599ff.). Drawing on arguments by Glenberg and Robertson (1999), Barsalou (1999a) posited that language comprehension must be viewed as preparation for situated action. He argues this holds for both present and displaced entities, for familiar and novel situations. Stronger grounding effects are predicted for immediate situations than those witnessed recently and even less pronounced effects for situations that were encountered a long time ago or that are entirely unfamiliar (p. 73). Zwaan (2014) used a related idea to predict grounding to vary by situation, with strong effects for referential and instruction situations (e.g., instructing someone to bring an object that is not present), and reduced effects when there is no overlap between the current situation and what language is about (e.g., as is the case for scientific articles). Predicting grounding effects via a characterization of the context is also the focus in the framework by Myachykov, Scheepers, Fischer, and Kessler (2014). They distinguish between invariant (e.g., gravity, p. 446) and less invariant dimensions of the world (e.g., the current situation and goals of an agent). Stronger grounding effects are predicted for less variable dimensions. Relatedly, Knoeferle and Crocker (2007, p. 542) predicted that the importance of scenes for comprehension would vary depending on the extent of referential success as well as locational or temporal cues in the utterance that clarify the (ir)relevance of the immediate scene (e.g., someone in an adjacent room calls out *The cat has jumped on the table again*, and at the same time you are in the TV room watching a commercial featuring a cat; temporary reference may occur but you quickly realize that your friend is talking about another cat, presumably reducing the relevance of the TV-cat.).

Other factors that can modulate grounding effects are how easily integrable linguistic and non-linguistic stimuli are Kaschak et al., 2005). ‘Integrable’ means that language-based representations derived from the mention of *a car* can be integrated with representations of an object like a car but not with representations of an unrelated object like a spiral. If they are integrable (as in hearing *The car approached you* while seeing a car approaching), processing should be faster for motion direction matches compared with mismatches (when the car was shown as driving away; reflected in sentence sensibility judgement latencies). But when concurrent object perception (e.g., a spiral creating motion towards or away) is non-integrable with language (e.g., *The car approached you*), integrability predicts slower processing for direction-matching than mismatching pairs (for a further account see Connell & Lynott, 2012; Aravena et al., 2012, for related evidence on language-guided modulation of grip force).
